# Automatic Detection of Fatigued Gait Patterns in Older Adults: An Intelligent Portable Device Integrating Force and Inertial Measurements with Machine Learning

**DOI:** 10.1007/s10439-024-03603-z

**Published:** 2024-08-13

**Authors:** Guoxin Zhang, Tommy Tung-Ho Hong, Li Li, Ming Zhang

**Affiliations:** 1 Department of Biomedical Engineering, Faculty of Engineering, The Hong Kong Polytechnic University, Hong Kong SAR, 999077 China; 2 School of Fashion and Textiles, The Hong Kong Polytechnic University, Hong Kong SAR, 999077 China; 3 Research Institute for Sports and Technology, The Hong Kong Polytechnic University, Hong Kong SAR, 999077 China

**Keywords:** Fatigued gait patterns, Older adults, Intelligent portable device, IMU, Machine learning

## Abstract

**Purpose:**

This study aimed to assess the feasibility of early detection of fatigued gait patterns for older adults through the development of a smart portable device.

**Methods:**

The smart device incorporated seven force sensors and a single inertial measurement unit (IMU) to measure regional plantar forces and foot kinematics. Data were collected from 18 older adults walking briskly on a treadmill for 60 min. The optimal feature set for each recognition model was determined using forward sequential feature selection in a wrapper fashion through fivefold cross-validation. The recognition model was selected from four machine learning models through leave-one-subject-out cross-validation.

**Results:**

Five selected characteristics that best represented the state of fatigue included impulse at the medial and lateral arches (increased, *p* = 0.002 and *p* < 0.001), contact angle and rotation range of angle in the sagittal plane (increased, *p* < 0.001), and the variability of the resultant swing angular acceleration (decreased, *p* < 0.001). The detection accuracy based on the dual signal source of IMU and plantar force was 99%, higher than the 95% accuracy based on the single source. The intelligent portable device demonstrated excellent generalization (ranging from 93 to 100%), real-time performance (2.79 ms), and portability (32 g).

**Conclusion:**

The proposed smart device can detect fatigue patterns with high precision and in real time. *Significance:* The application of this device possesses the potential to reduce the injury risk for older adults related to fatigue during gait.

## Introduction

Neuromuscular fatigue implicates the performance of muscle control and the coordination of multiple joints [1], leading to a deterioration in gait balance [2–4] and stability [5]. The decline in gait performance heightens the risk of injuries such as falls, which are a leading cause of injury and the economic burden among older adults [6]. Real-time detection of neuromuscular fatigue is crucial for implementing effective interventions to reduce the probability of such injuries. While laboratory equipment like motion capture systems and force plates offer high accuracy for motion analysis, they are not suitable for personal use in outdoor environments. Therefore, identifying gait patterns associated with neuromuscular fatigue using portable sensors is the most practical approach for daily activities and exercise.

Among the many wearable devices used to evaluate neuromuscular fatigue, surface electromyography (EMG) [5, 7] , muscle oxygen saturation (SmO_2_) [8, 9], inertial measurement unit (IMU) [10–15], and plantar force sensors [16–18] are the most common. However, surface EMG sensors are sensitive to skin cleanliness and placement, and their repeatability and portability are suboptimal. Measurements of SmO_2_ such as those obtained through near-infrared spectroscopy are sensitive to movement and unsuitable for assessing long-term dynamic motion. In contrast, IMUs and plantar force sensor are highly robust. IMUs can be conveniently attached to the body (such as the lower back, ankle, and shank) or shoes [13, 15], and force sensors can be placed under the foot soles. Moreover, IMUs and force sensors have minimal impact on normal gait.

Neuromuscular fatigue during walking diminishes the lower extremity’s cushioning ability, leading to a more significant impact when the foot strikes the ground [19, 20], which can be measured by the IMU. Fatigue also alters plantar pressure distribution as pronation increases after the fatigue of the plantar intrinsic foot muscles [19, 21, 22], which can be measured by plantar force sensors [16, 17]. Therefore, it is a promising solution to detect fatigue by measuring plantar loading with force sensors and foot dynamics with the IMU. Combining biomechanical information with machine learning is the current trend in research on exercise and health monitoring. In the recognition of fatigue gait pattern during walking, SVM is the most widely used model due to its suitability for small samples and strong predictive power [11–14], followed by long short-term memory model [10]. Previous studies have incorporated biomechanical data such as segment kinematics [10–14] and plantar loading [23], along with machine learning techniques including support vector machines [11–14, 23] and long short-term memory models [10], to achieve fatigue gait recognition in walking tasks, with accuracies ranging from 62.5 to 95.71%. While previous studies have measured fatigue using various body parts, we posit that the foot's position as the most distal segment from the body's center and its initial contact with the ground makes it a prime candidate for early detection of lower limb muscle fatigue. Consequently, we hypothesize that employing an IMU to capture foot kinematics and multiple pressure sensors to gauge plantar load, in conjunction with a machine learning algorithm, will yield a more effective method for fatigue detection.

In this study, we aimed to examine the validity of early detecting the fatigued gait pattern in older adults through a development of smart portable device that combines portable sensors and a machine learning model.

## Materials and Methods

**Fig. 1:**
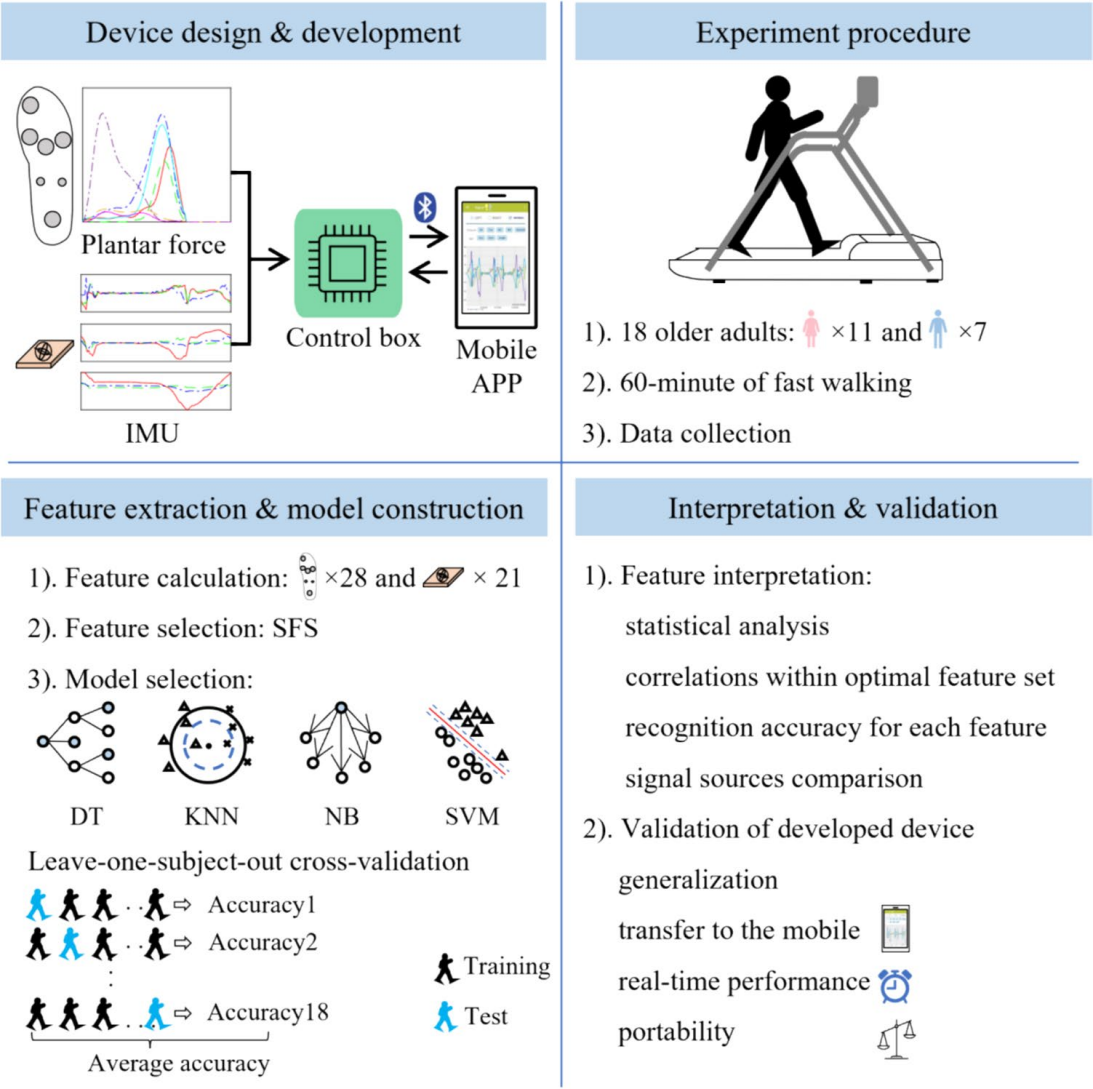
Research framework

The research framework was composed of four parts (Fig. 1). First, a smart device was designed and developed to measure the foot’s kinetic and kinematic using a custom plantar regional force insole and a single IMU. An experiment was then conducted to collect data, followed by the determination of feature set and recognition model. At last, the selected features were interpreted, and the smart device’s performance was verified.

### Device Development

**Fig. 2:**
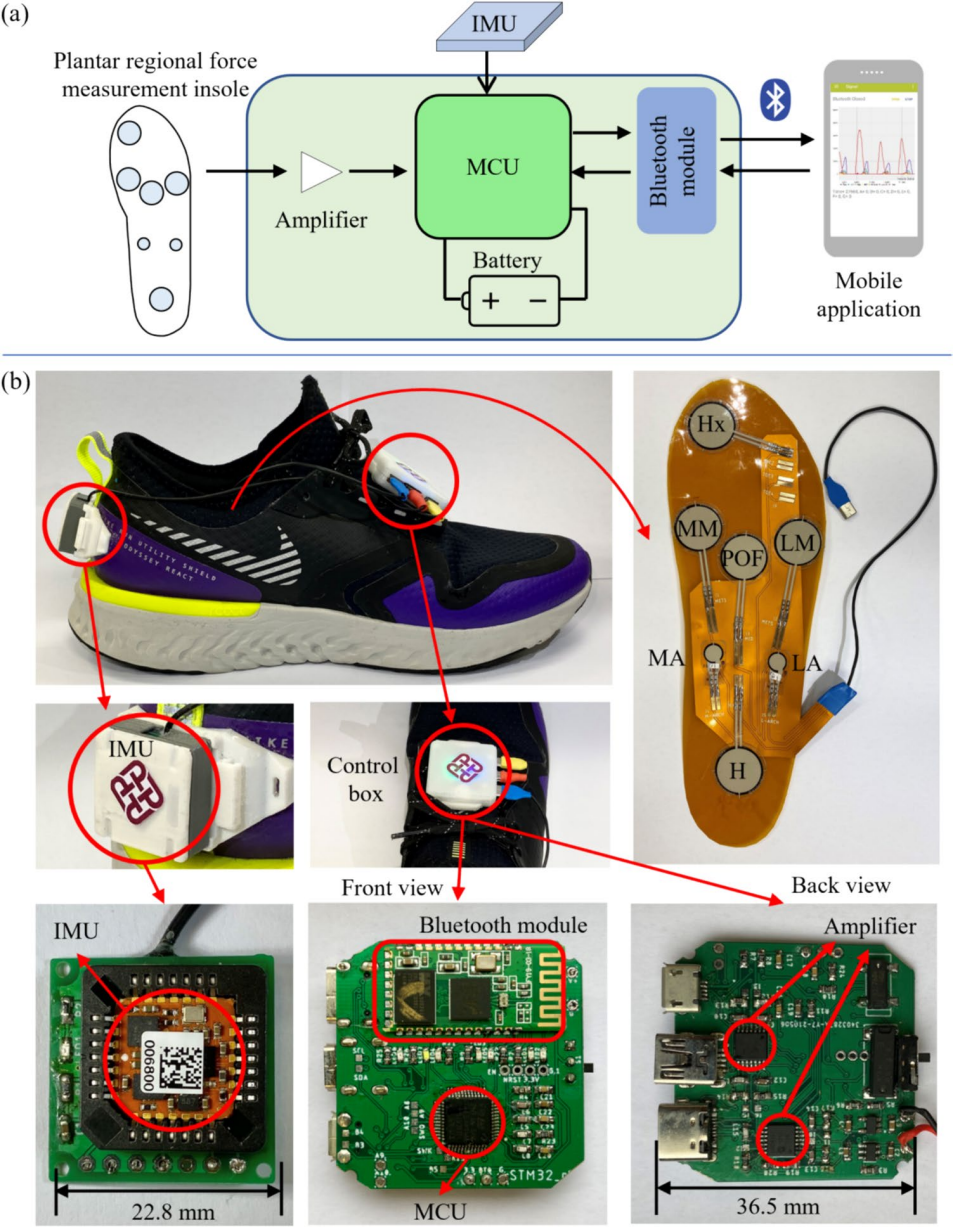
The design and development schematic of the developed device

This smart device measured the foot’s kinetics and kinematics using a custom plantar regional force measurement insole and a single IMU (Fig. 2a). After preliminary processing by the microcontroller unit (MCU), the data obtained by force and IMU sensors were wirelessly transferred to a custom Android application via a Bluetooth module. This custom mobile application, which integrated the LIBSVM toolbox [24], was developed using a commercial integrated development environment (Android Studio 4.2.2, JetBrains, Prague, Czech Republic; Google, Menlo Park, California, USA). LIBSVM is a mature toolbox that integrates the SVM in multiple code sources and can help developers use the SVM algorithm more conveniently. The mobile application was used to collect, display, analyze, and recognize fatigued gait patterns in real time.

The custom insole (Fig. 2b) incorporated seven thin-film standard flexible force sensors (FlexiForce A301 & A401, Tekscan Inc., Boston, Massachusetts, USA) to collect plantar force at the hallux, medial and lateral metatarsal, posterior of the footpad, medial and lateral arch, and the heel regions, based on our previous studies [15–17]. The insole’s plantar force measurement layer was covered with a thin, soft layer. A nine-axis IMU (MTi-7-T, Xsens Technologies B.V., Enschede, Netherlands) was used to collect the triaxial acceleration, angular velocity, and rotation angle of the rearfoot [15]. IMU was connected to the control box via a socket and fixed to the posterior heel position of the right shoe.

The control box integrated an MCU (STM32L051R6T6, STMicroelectronics, Geneva, Switzerland), a Bluetooth module (HC-05, Maker Factory Bluetooth, Shanghai, China), two amplifiers (MCP6004-I/ST, Microchip Technology Inc., Arizona, USA), a rechargeable lithium battery, and a printed circuit board to enhance integration and connection stability [25]. As depicted in the hardware installation diagram, the custom insole and IMU were connected to a control box attached to the bottom of the shoe tongue’s upper surface to minimize vibration impact.

### Participants

Eighteen older adults (11 females and 7 males, 63.4 ± 4.1 years, 159.0±7.5 cm, and 60.3± 9.7 kg) were recruited for this study. All participants were independent walkers without any neuromuscular abnormalities, pains (e.g., knee pain), or diseases that could potentially affect their walking patterns. Individuals with obesity (BMI > 30 kg/m^2^) or a history of falls within the past six months were excluded from the study. The study received approval from the University Human Subjects Ethics Sub-Committee (No: HSEARS20190919001). Each participant was thoroughly informed about the study and provided written consent prior to participation. The sample size was determined using G*Power 3.1.9.7 (Universität Düsseldorf, Düsseldorf, Germany) [26], with an estimated 18 participants based on a significance level of 0.05, statistical power of 0.8, and a medium effect size of Cohen’s *f* = 0.32 [27] using the *F*-test within factor with two repeated measures as this study compared gait parameters before and after fatigue using the Wilcoxon signed-ranks tests.

### Procedures

The experiments were conducted on a treadmill (Unisen Inc., Tustin, California, USA). Prior to the 60-min walking trial, each participant completed a 5-min acclimation walk [28]. There was no interruption between the acclimation walk and the walking trials. During the acclimation walk, the comfortable fast walking speed was determined, with an average speed of 3.83 ± 0.52 km/h. Participants completed the walking trials at their individual speeds, wearing uniform clothes, socks, and a specific type of neutral running shoes (BQ1671-002, Nike Inc., Beaverton, USA) to minimize the influence of clothing differences. Plantar regional force and IMU data were simultaneously collected using a custom smart device at a sampling rate of 110 Hz during the 60-min walking trial.

### Feature Extraction

All features were calculated based on the gait cycle from the right foot, with the gait cycle identified using a threshold of 10 N [29]. The outcome variables included 28 variables based on force sensors and 21 variables based on IMU sensor (Table 1). The coefficient of variation (CV) is defined as the standard variation divided by the mean absolute value. The jerk is the first derivative of acceleration with respect to time, it measures the rate of change of acceleration. The rotation angle was defined as the angle of the foot relative to the stationary stance phase. The IMU is prone to drift in the vertical axis angle (*θ*_Tra_), which tends to worsen over time. By utilizing the relatively stationary state of the foot during the flat-foot phase, we can correct for this drift without concern for the IMU's vertical axis angle. Therefore, we subtract the average angle during the flat-foot phase (*θ*_Flat-foot_) from each gait cycle to obtain the foot's rotation angle relative to this period, calculated as

1 $$ \theta_{{{\text{Flat-foot}}}} = \frac{1}{{n_{15\% - 25\% } }}\mathop \sum \limits_{i = 15\% }^{i = 25\% } \theta_{{{\text{Tra}}}} \left( i \right) $$

2 $$ \theta_{{{\text{Tra}}}} \left( i \right) = \theta_{{{\text{Tra}}}} \left( i \right) - \theta_{{{\text{Flat}} - {\text{foot}}}} $$

**Table 1.** Outcome variables

Sensors	Variables
Force	contact time (CT), peak force (PF), impulse (Imp), and the coefficient of variation of force (FCV) at seven regions: CT_Hx_, CT_MM_, CT_POF_, CT_LM_, CT_MA_, CT_LA_, CT_H_; PF_Hx_, PF_MM_, PF_POF_, PF_LM_, PF_MA_, PF_LA_, PF_H_; Imp_Hx_, Imp_MM_, Imp_POF_, Imp_LM_, Imp_MA_, Imp_LA_, Imp_H_; FCV_Hx_, FCV_MM_, FCV_POF_, FCV_LM_, FCV_MA_, FCV_LA_, FCV_H_
IMU	maximum (Max), root mean square (RMS), and CV of the resultant acceleration (*acc*_Res_), resultant jerk (*j*_Res_), resultant angular velocity (*ω*_Res_), and resultant angular acceleration (*α*_Res_): *acc*_Res, Max_, *acc*_Res, RMS_, *acc*_Res, CV_; *j*_Res, Max_,* j*_Res, RMS_,* j*_Res, CV_; *ω*_Res, Max_, *ω*_Res, RMS_, *ω*_Res, CV_; *α*_Res, Max_, *α*_Res, RMS_, *α*_Res, CV_ maximum, range, and CV of the angle in the sagittal (Sag), coronal (Cor), and transverse (Tra) planes: *θ*_Sag, Max_, *θ*_Sag, RMS_, *θ*_Sag, CV_ *θ*_Cor, Max_, *θ*_Cor, RMS_, *θ*_Cor, CV_ *θ*_Tra, Max_, *θ*_Tra, RMS_, *θ*_Tra, CV_

*H* heel; *Hx* hallux; *LA* lateral arch; *LM* lateral metatarsal; *MA* medial arch; *MM* medial metatarsal; *POF* posterior of footpad.

Because the corresponding moments of the foot flat and heel off events are approximately 10 and 30% of the gait cycle [30], 15–25% of the gait cycle was adopted as the flat-foot period.

All offline data processing and statistical analyses were completed using custom codes, the Signal Processing Toolbox, and the Statistics and Machine Learning Toolbox on MATLAB 2023a (MathWorks Inc., Natick, Massachusetts, USA).

### Machine Learning and Validation

#### Dataset

Previous studies have shown that 60 min of fast walking can induce fatigued gait pattern in older adults, as evidenced by increased self-perceived fatigue levels and gait variability, and decreased activity of major lower limb muscles and gait stability [15–17, 31, 32]. In this study, the first and last 5 min were hypothesized to represent non-fatigue and fatigued states, respectively. Each sample was 10 s long. Using a sliding window with an 8-s overlap, each state yielded 150 samples (5 × 60 ÷ (10 − 8)). Therefore, the dataset comprised 5400 samples (150 samples/state × 2 states × 18 participants).

#### Recognition Model

The optimal feature set for each machine learning model was determined using forward sequential feature selection in a wrapper fashion, which selects a feature subset by adding features sequentially until the selection criteria is met. The selection criteria were defined as the misclassification rate using fivefold cross-validation no longer decreased when new features were added. The misclassification rate refers to the number of misclassified samples divided by the total number of samples. *K*-fold cross-validation, also named *k*-fold rotation estimation, first divides the sample into *k* subsets, then uses one subset as the test set, and the remaining *k − *1 subsets as the training set. After *k* times of looping, *k* models and their corresponding errors are obtained. The average of these *k* errors is used as the cross-validation error. Because the test set has unknown data in each test, cross-validation is a good measure of the true predictive power of the model and can effectively prevent overfitting.

The optimal recognition model was selected from four machine learning models: Naïve Bayes (NB), k-nearest neighbor (KNN), decision tree (DT), and support vector machine (SVM). The selection criteria for the classification model were defined as the minimal misclassification rate. The misclassification rate of each recognition model was obtained using its corresponding optimal feature set through leave-one-subject-out cross-validation (LOSOCV), as shown in the lower left corner of Fig. 1. LOSOCV is like k-fold cross-validation. The difference is that* k*-fold cross-validation randomly divides all samples of all participants into subsets, while LOSOCV divides subsets based on participant (each test set is all the samples of a certain person). LOSOCV was adopted as it can minimize the bias of recognition accuracy. The four models used the same loss function and optimizer: the minimal expected misclassification cost and the Bayesian optimization.

#### Interpretation and Validation

To interpret the selected features, each feature in the optimal feature set was compared before and after fatigue using the Wilcoxon signed-ranks tests with Bonferroni correction at a significance level of* α* < 0.05. The importance of each feature was investigated by calculating its identification accuracy using the selected recognition model. The effect of signal source and correlations between features within the optimal feature set were also examined. Finally, the codes for data processing and pattern recognition were transferred to a mobile phone. The generalization, real-time performance, and portability of the smart device were verified.

## Results

### Optimal Feature Set

**Fig. 3:**
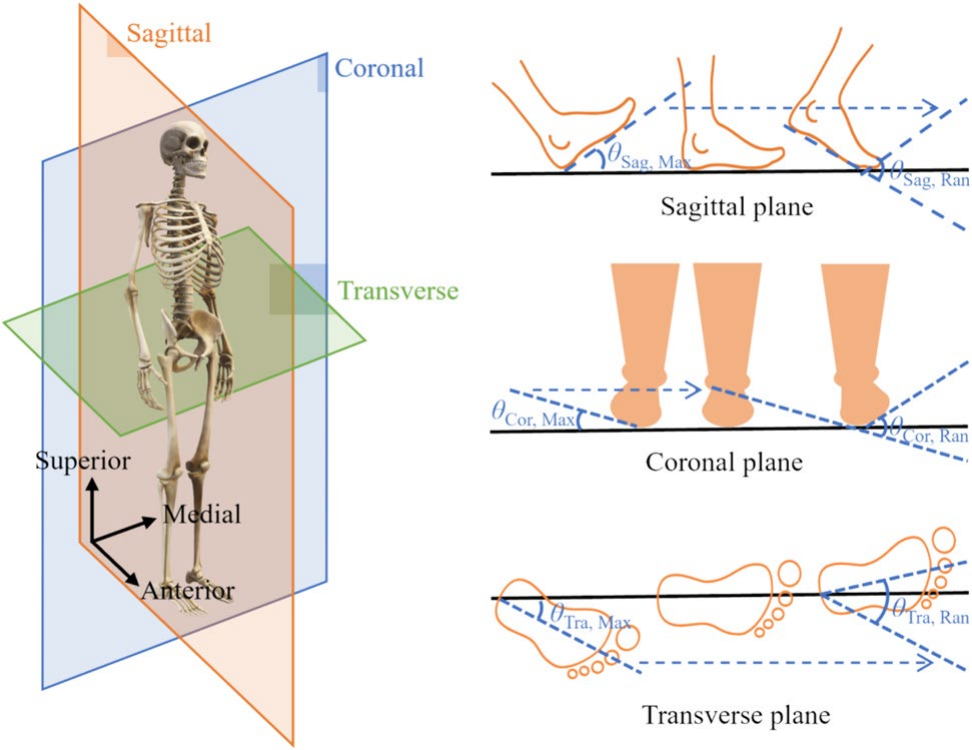
Schematic diagram of coordinate system and angle-related parameters

A total of ten features were selected using forward sequential feature selection, five of which were based on the plantar regional force signal and five on the IMU signal (Fig. 3a). Compared to the non-fatigue state, six features in the fatigued state showed significant differences. These differences included the impulse at the medial and lateral arch (*p* = 0.002 and *p* < 0.001), the RMS and CV of resultant angular acceleration at the posterior heel (*p* = 0.035 and *p* < 0.001), and the maximum and range of rotation angle in the sagittal plane at the posterior heel (*p* < 0.001 and *p* < 0.001). The recognition accuracy based on each feature of the optimal feature set was investigated (Fig. 3b). Five features achieved over 80% recognition accuracy, including the impulse at the lateral and medial arch region (92 and 83%, respectively), the maximum and range of rotation angle in the sagittal plane at the posterior heel (89 and 88%, respectively), and the CV of resultant of angular acceleration at the posterior heel (80%).

### Machine Learning

**Fig. 4:**
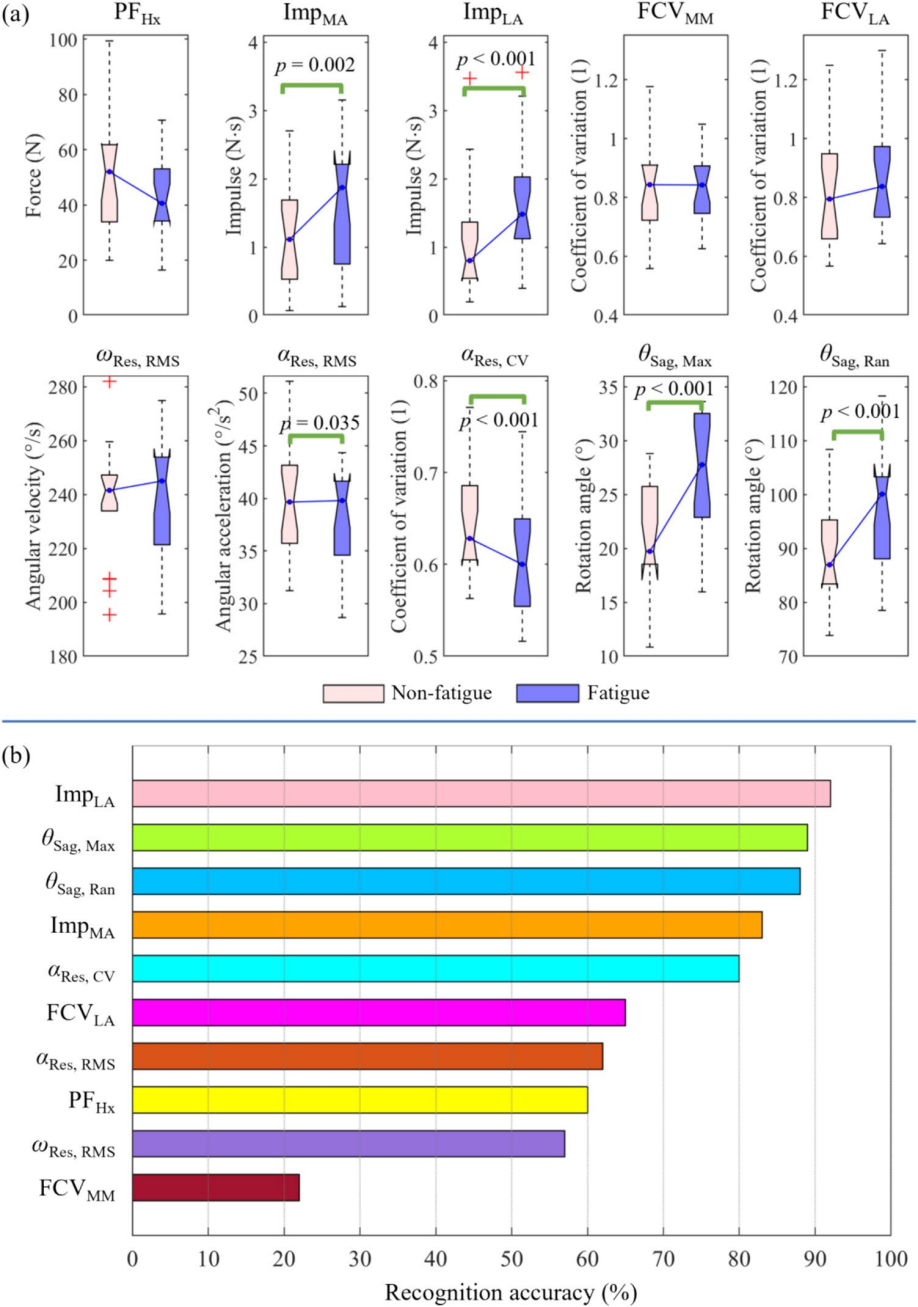
Statistical results and recognition accuracy based on each single feature. Abbreviation: CV = coefficient of variation; FCV = the CV of force; Hx = hallux; Imp = impulse; MA = medial arch; Max = maximum; MM = medial metatarsal; LA = lateral arch; PF = peak force; Ran = range; Res = resultant; RMS = root mean square; Sag = sagittal

In this study, SVM outperformed the other three machine learning models, achieving a recognition accuracy of 99% for both non-fatigue and fatigue states (Fig. 4a). NB and KNN also performed well, with an average accuracy of 96%. The recognition accuracy based on dual signals was higher than that based on a single signal, either plantar force or IMU, 99 vs. 95% (Fig. 4b). Most correlation coefficients between features in the optimal feature set were small or medium (Fig. 4c). The correlation coefficient was measured using the Pearson correlation coefficient *r*, where *r* greater than 0.5 is regarded as a strong correlation [33].

### Validation

**Fig. 5:**
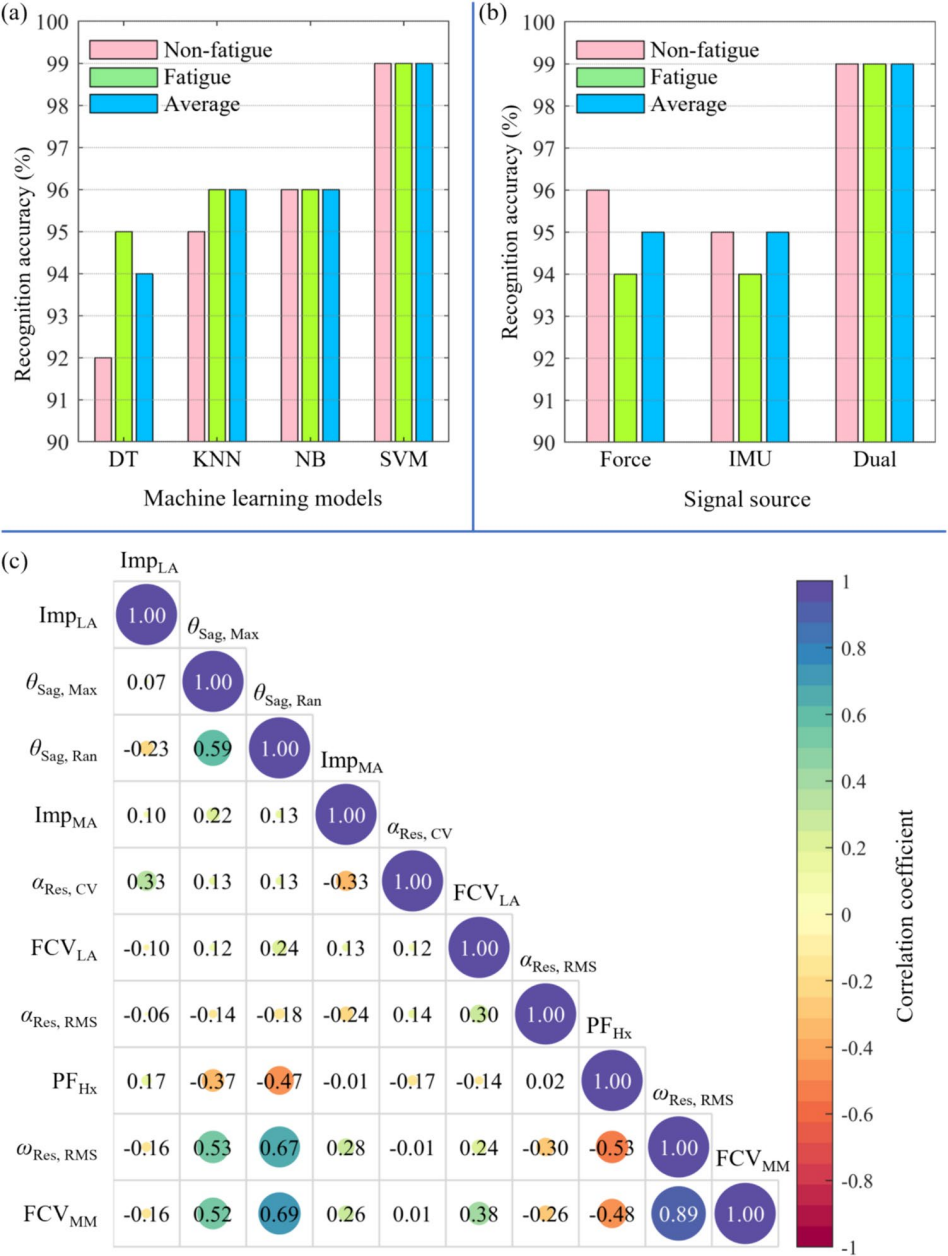
The comparison of different models and signal source, and the correlation coefficient between features in the optimal feature set

The generalization of the proposed smart device was assessed using LOSOCV. The recognition accuracy of each participant ranged from 93 to 100% (Fig. 5a). The total time consumption, including data processing and fatigue recognition, was only 2.79 ms (Fig. 5b). The total weight of the smart device developed in this study was 32 g (Fig. 5c).

## Discussion

**Fig. 6:**
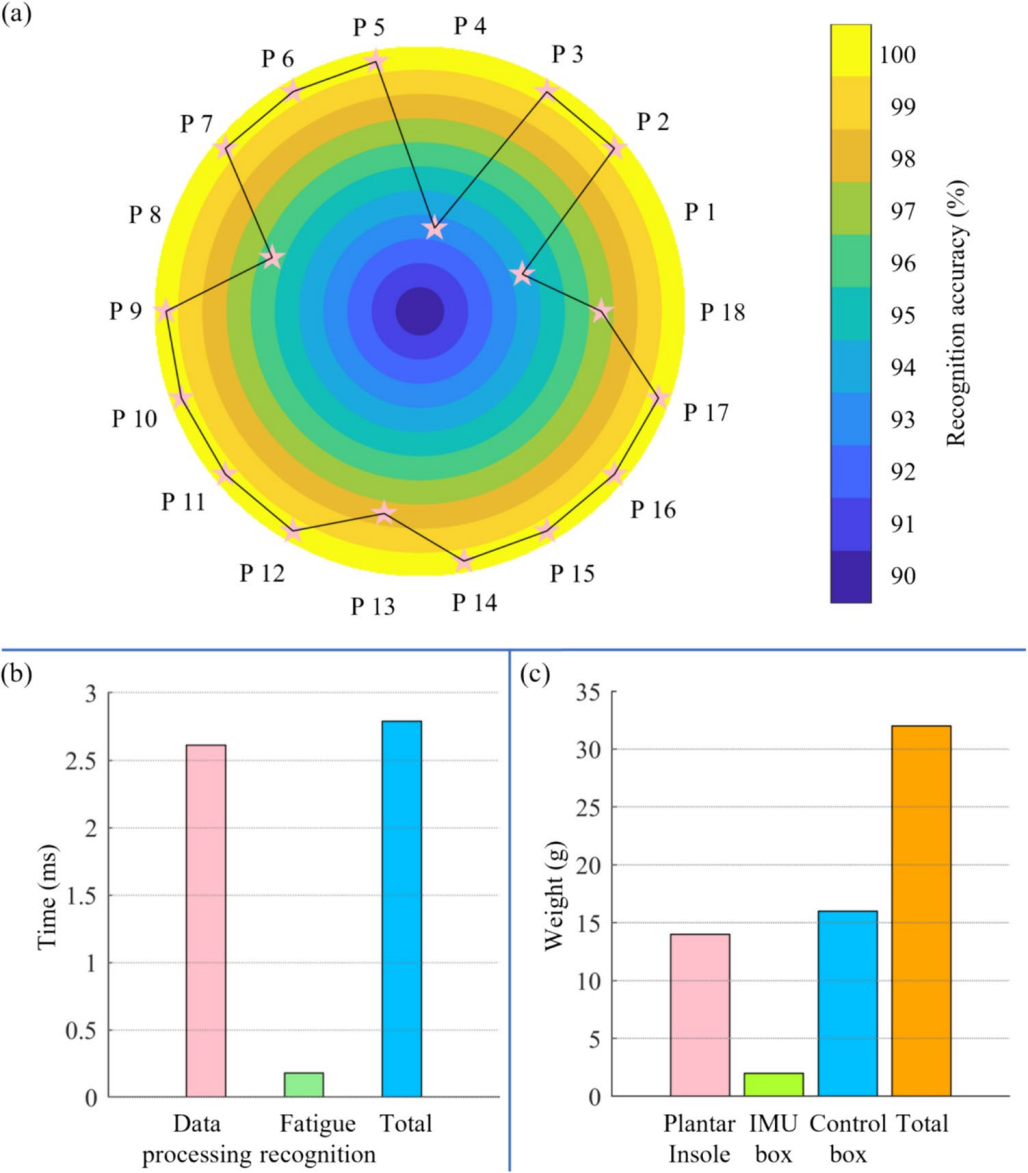
The validation of the developed smart device: generalization, real-time performance, and portability

This study examined the validity of early detecting fatigued gait patterns in older adults through a developed smart portable device. The fatigue effect was significant manifested in both gait loading and swing patterns, as measured by seven force sensors and a single IMU. The smart device developed in this study demonstrated high identification accuracy (99%), excellent real-time performance (2.79 ms), and portability (32 g) (Fig. 6).

Fatigue resulted in a significantly larger maximum angle in the sagittal plane, which could imply a higher risk of falls due to the increased likelihood of slipping with a larger foot-floor contact angle [34–37]. After 60 min of brisk walking, the rotation angle range in the sagittal plane also increased, possibly due to its high correlation with the maximum angle in the sagittal plane. The resultant angular acceleration and its coefficient of variation significant decreased compared to non-fatigue conditions, potentially due to muscle control strategies that pre-adjusted to reduce vibration and impact force before the heel strike [38]. The parameters of the swing phase, including the maximum angle and rotation range of angle in the sagittal plane, and the resultant angular velocity and its coefficient of variation, all demonstrated a high fatigue classification rate. This is likely because gait parameters before foot-ground contact play a crucial role in maintaining postural balance [34]. The impulses recorded at the medial and lateral arches in this study were notably lower than those reported in previous research [16, 20]. This discrepancy can be attributed to the smaller sensor area used in our study. For instance, in our prior work [16], the medial and lateral arch areas were approximately 2000 mm^2^, resulting in measured impulses around 30 Ns. In contrast, the current study utilized a sensor area of merely 71 mm^2^ for these regions, with corresponding impulses of about 1 Ns. Thus, both the arch area and the measured impulses in this study are roughly 1/30 of those in the previous study. The impulse at the arch region significantly increased after fatigue, typically associated with increased pronation foot [20] and decreased arch height [16]. These changes are indicative of muscle fatigue in the lower extremities [19, 21, 22] and could explain the high accuracy of fatigue identification based on impulse at the medial or lateral arch.

The recognition accuracy based on plantar regional forces in this study (95%) was significantly higher than that of ground reaction force (GRF) in previous studies [23]. This could be due to the more comprehensive information provided by multiple plantar regional forces compared to a single GRF. The recognition accuracy based on a single IMU in this study was 95%, which is higher than that reported in previous studies [12, 14]. This could be attributed to the fact that previous studies attached the IMU to the lower back, thigh, or shank, rather than the foot [12, 14, 39, 40]. Given that the foot is further from the body’s center and lacks the cushioning of joints, the impact of fatigue on the foot tends to be more pronounced. Specifically, the heel appears to be more indicative of gait patterns than the toes, as it is the first part of the foot to contact the ground and absorb impact [39]. Interestingly, while recognition accuracy can be enhanced by utilizing multiple IMUs [10, 11, 13] or plantar regional forces, the accuracy based on a single source was still lower than that based on dual signal sources. This finding aligns with the principles of multimodal learning theory [41].

The smart device developed in this study demonstrated excellent generalization, with participant identification accuracy ranging from 93 to 100%. The total time for data processing and pattern recognition was less than 3 ms, well within the normal gait cycle, making it suitable for real-time needs. The total weight of the smart device was approximately 30 g, significantly lighter than a pair of regular shoes, which typically weigh around 500 g. This lightweight design ensures that the device does not affect the walking task, as the metabolic cost only increases approximately by 1% per 100 g of mass added to each shoe [42, 43]. Unlike other devices that needed to be attached to various body parts, this smart device is affixed to the shoe, eliminating the potential influence of varying installation positions on the smart device's performance with each use [10]. Its lightweight and installation-free effectively minimize the device’s impact on normal gait patterns during walking.

This study has some limitations. It assumed that older adults would experience fatigue after long distance brisk walking, which may not always be the case, despite the observed increase in self-perceived fatigue levels and decreased activity of major lower limb muscles and gait performance [15–17, 31, 32]. The experiments were conducted on a treadmill at a fixed speed, without considering the variations in speed, inclines, declines, and turns that occur during daily walking. The forward sequential feature selection method was employed to optimize the most effective set of features, but several features are strongly correlated. Future studies should consider these factors, as well as the optimization of the number and location of force sensors in the custom insole.

In conclusion, this study developed a portable smart device, equipped with seven force sensors and a single IMU, to assess the feasibility of early detecting neuromuscular fatigued gait pattern in older adults. The features most sensitive to fatigue were the increased impulse at medial and lateral arches, the increased contact angle and rotation range of angle in the sagittal plane, and the decreased variability of the resultant swing angular acceleration. While the recognition accuracy based on a single signal source, either IMU or plantar force, was 95%, it increased to 99% when both signal sources were used. The smart device demonstrated excellent fatigue identification capabilities, real-time performance, and portability. The recognition accuracy through LOSOCV reached 99%, the processing time was less than 3 ms, and the weight was only 32 g.
